# Epigenome-wide association of neonatal methylation and trimester-specific prenatal PM_2.5_ exposure

**DOI:** 10.1097/EE9.0000000000000227

**Published:** 2022-10-03

**Authors:** Milan N. Parikh, Cole Brokamp, Erika Rasnick, Lili Ding, Tesfaye B. Mersha, Katherine Bowers, Alonzo T. Folger

**Affiliations:** a Division of Biostatistics and Epidemiology, Cincinnati Children’s Hospital Medical Center, Cincinnati, Ohio; b Department of Pediatrics, University of Cincinnati College of Medicine, Cincinnati, Ohio; c Division of Asthma Research, Cincinnati Children’s Hospital Medical Center, Cincinnati, Ohio

**Keywords:** DNA methylation, Fine particulate matter, Prenatal exposure

## Abstract

Exposure to particulate matter with an aerodynamic diameter smaller than 2.5 microns (PM_2.5_) can affect birth outcomes through physiological pathways such as inflammation. One potential way PM_2.5_ affects physiology could be through altering DNA methylation (DNAm). Considering that exposures during specific windows of gestation may have unique effects on DNAm, we hypothesized a timing-specific association between PM_2.5_ exposure during pregnancy and DNAm in the neonatal epithelial-cell epigenome.

**Methods:**

After collecting salivary samples from a cohort of 91 neonates, DNAm was assessed at over 850,000 cytosine-guanine dinucleotide (CpG) methylation sites on the epigenome using the MethylationEPIC array. Daily ambient PM_2.5_ concentrations were estimated based on the mother’s address of primary residence during pregnancy. PM_2.5_ was averaged over the first two trimesters, separately and combined, and tested for association with DNAm through an epigenome-wide association (EWA) analysis. For each EWA, false discovery rate (FDR)-corrected *P* < 0.05 constituted a significant finding and every CpG site with uncorrected *P* < 0.0001 was selected to undergo pathway and network analysis to identify molecular functions enriched by them.

**Results:**

Our analysis showed that cg18705808 was associated with the combined average of PM_2.5_. Pathway and network analysis revealed little similarity between the first two trimesters. Previous studies reported that *TMEM184A*, the gene regulated by cg18705808, has a putative role in inflammatory pathways.

**Conclusions:**

The differences in pathway and network analyses could potentially indicate trimester-specific effects of PM_2.5_ on DNAm. Further analysis with greater temporal resolution would be valuable to fully characterize the effect of PM_2.5_ on DNAm and child development.

**What this study adds**

This study expands on previous research by presenting evidence for an effect of prenatal exposure to particulate matter with an aerodynamic diameter less than 2.5 microns (PM_2.5_) on neonatal DNA methylation (DNAm) that varies based on the timing of the exposure. If these findings can be validated with larger sample sizes and higher temporal resolution, they may provide information on windows during which neonatal DNAm is sensitive to change by prenatal PM_2.5_ exposure. We have also identified that PM_2.5_ exposure is associated with DNAm at a locus within the body of gene *TMEM184A*, which has been implicated in inflammatory pathways.

## Introduction

A wide variety of environmental exposures can affect fetal development. One exposure of interest is ambient air pollution, specifically particulate matter with an aerodynamic diameter less than 2.5 microns (PM_2.5_). Increased ambient PM_2.5_ has adverse effects on human health, including mortality and morbidity related to respiratory, cardiovascular, and neurobehavioral health, through activation of inflammatory pathways.^1–6^ Neonatal outcomes are also negatively impacted by PM_2.5_, with high exposures during fetal development having associations with low birth weights, abnormal lung development, and increased risk of preterm birth.^7–11^ Increased PM_2.5_ exposures later in childhood have been associated with increased pediatric psychiatric emergency department utilization and increased risk for childhood cancer.^12,13^ While the association between PM_2.5_ and these health outcomes is well established, the underlying mechanisms remain unclear. One potential pathway linking PM_2.5_ exposure to long-term health outcomes is DNA methylation (DNAm), an epigenetic mechanism that is responsive to the environment and impacts health through changes in gene regulation.^14^

DNA methylation is the process in which methyltransferases bind methyl groups to cytosine bases to form 5-methylcytosines, which can have a regulatory function by silencing gene transcription.^15^ Cytosine-guanine dinucleotides (CpGs) are the most frequent sites of DNAm, and changes in DNAm at these sites can have functional consequences to biological processes and, therefore, health.^16,17^ DNAm, which is present in the genome from conception, is also mutable in the face of environmental factors, meaning that the transcription of the genome can change in response to exposures in the external and fetal environments.^18–23^ Therefore, identifying exposures that are associated with DNAm changes could suggest a mechanism for short- or long-term encoding of those exposures into the epigenome. In fact, altered DNAm associated with PM_2.5_ levels has been identified in specific genes in several populations.^24–26^ As these methylation changes to the DNA could lead to adverse health effects, characterizing the relationship between exposures like fine particulate matter air pollution and DNAm can be a valuable step towards better understanding health and disease.

Exposure to exogenous environmental factors during pregnancy has been associated with DNAm changes at birth. For example, differing levels of socioeconomic status (SES) and community deprivation in the region of a child’s birth were associated with differential methylation of the infant’s epigenome at birth.^27,28^ In studies of air pollution, increased exposure to PM_2.5_ during pregnancy was associated with a decrease in global DNAm as well as a decrease in methylation of specific genes in placental DNA at birth.^29,30^ Changes in DNAm associated with prenatal exposure to PM_2.5_ can even be detected in salivary samples as late as at 15 years of age.^31^ Of particular note is a study utilizing a large multisite cohort that identified 14 CpG sites at which infant DNAm was associated with prenatal PM_2.5_ exposure averaged across pregnancy.^32^ While averaging exposures across pregnancy does provide a robust and powerful marker of total PM_2.5_ exposure during pregnancy, it is important to consider that the impact of an exposure on a specific health outcome may depend on the timing of that exposure.^16,30^ Given the sequential progression of anatomical and physiological structures during fetal development, exposures during specific windows of gestation may have unique effects on DNAm, depending on the phase of development occurring during that window.^33,34^ We hypothesized a timing-specific association between PM_2.5_ exposure during pregnancy and differential methylation at specific CpG sites in the neonatal epigenome. Our objectives were to (1) conduct an epigenome-wide association study (EWAS) and identify differentially methylated CpG sites in neonates that have DNAm values related to ambient PM_2.5_ exposure levels during three different windows during pregnancy and (2) determine the biological networks and pathways enriched by the top CpG sites from the EWAS through pathway and network analysis to characterize potential downstream physiological effects of PM_2.5_ exposure during the three windows of pregnancy.

## Methods

### Participants

This study combined participants from two related studies that enrolled from the same sampling frame, an ongoing home visiting program in Cincinnati, Ohio called Every Child Succeeds. Inclusion criteria for the program included that mothers were living in poverty, assessed through metrics such as income under 300% of the federal poverty level or receiving Medicaid.^35^ The Pregnancy and Infant Development (PRIDE) study was initiated as a pilot study in 2014, enrolling 53 mother-infant dyads, and was restarted in 2018 with a goal of enrolling 375 additional pairs.^35,36^ The PRIDE study enrolls pregnant women and follows the offspring postnatally at 1, 4, 12, and 18 months (follow-up in the pilot study was 1 month). Additional dyads were participants in a BioBank study (ECSBio) that collected specimens at 1 month and obtained similar exposure data. Pregnant women were enrolled between 2015 and 2019, and 93 have been followed through 1 month postnatal. The studies were approved by the Cincinnati Children’s Hospital Medical Center Institutional Review Board (IRB approval numbers: 2019-0808 and 2019-0588); all participants provided written informed consent.

### Measures

Maternal demographics, including age and race, were collected at the first study visit (prenatal visit for PRIDE and 1-month postnatal for ECSBio). Additionally, the primary address of residence during pregnancy was collected as part of the study.

### Air pollution exposure assessment

We geocoded each dyad’s primary residential address using a previously validated custom address range geocoder.^37^ Daily average PM_2.5_ concentrations were estimated at a resolution of 0.75 sq km using a previously validated machine learning model that used data on regional PM_2.5_ concentrations and emissions sources, planetary boundary layer height, wind speed, air temperature, and other spatiotemporal characteristics trained on PM_2.5_ concentrations measured by the Environmental Protection Agency (EPA).^38^ The cross validated *R*^2^ for this model in our study region was 0.97 for annual exposures and 0.96 for monthly exposures. Daily predictions were averaged for each dyad’s first trimester, second trimester, and over both of the first two trimesters of pregnancy through a simple arithmetic mean. The end of the second trimester was selected as a convenient endpoint due to the variability in the timing of birth in the third trimester, which leads to different lengths of pregnancy. In utero and ex utero, air pollution exposures are highly distinct, so we chose not to continue the investigation into the third trimester where birth could occur at different times. This two-trimester average represents the cumulative exposure over this period, while the individual trimester averages consider two major windows of exposure.

### DNA methylation

DNA methylation (DNAm) was measured using salivary samples at a postnatal visit between 3 and 6 weeks of life. A total of 10 buccal swabs were collected from each neonate by swabbing with a sponge on the inner cheek until saturated and alternating between samples designated for DNA extraction (stored in lysate solution) and for cell spinning (stored in phosphate-buffered saline). The DNAGenotek OGR-250 infant saliva collection kits were used to collect cells for DNA extraction in the same manner as Folger et al.^36^ Genomic DNA went through sodium bisulfite (BS) conversion and was assayed using microarray technology in the Genomics, Epigenomics and Sequencing Core at the University of Cincinnati.^22,39^ BS-converted DNA was hybridized to the Illumina Infinium MethylationEPIC BeadChip, which assesses DNAm states at over 850,000 CpG sites in varied regions of the genome.

### Array processing

Array processing was performed in R (R Core Team; Vienna, Austria), specifically using the *minfi* package (version 1.36.0) to import methylation data and estimate DNAm intensity.^40^ First, raw signal intensities were read into R, then quality control was performed at the sample level using kernel density plots of beta values and Illumina controls. Prediction of sex from methylation patterns was used to identify discrepancies between predicted and reported sex indicative of poor sample quality. The quality of the methylation arrays were evaluated in R (R Core Team; Vienna, Austria) using the *ewastools* package (version 1.7) to assess Illumina’s 17 control metrics.^41^ All samples were within the limits for the Illumina controls.

CpG probes and sites also went through quality control. Probes/sites were excluded from analysis if they (1) had a detection *P* > 0.01; (2) had bead counts less than 3 in more than 5% of samples; (3) were on sex chromosomes; (4) had single nucleotide polymorphisms (SNPs) with a minor allele frequency of 5% or more or were within two positions of a SNP location; or (5) had probes with nonspecific cross-hybridization to other regions. A more stringent detection *P* value threshold was determined by comparing signals with a distribution derived from nonspecific background fluorescence to reduce the risk of spurious associations.^42^

Following quality control, raw intensity data were background-corrected and normalized using the normal-exponential with out-of-band probes within-array approach, a process that also included dye bias adjustment.^40,43,44^ Regression on Correlated Probes probe type adjustement for type I and II probes was performed in R (R Core Team; Vienna, Austria) using the *ENmix* package.^45^ β values (scores from 0 to 1 with 0 = unmethylated and 1 = methylated) and M values (logit transformation of β) were derived from normalized methylation intensities. Statistical analysis was performed on M values due to advantages over β values.^46^

### Cell type heterogeneity

To understand the heterogeneity of our cell samples, we estimated the proportions of cell types in samples from each of our participants using the *EpiDISH* package in R (R Core Team; Vienna, Austria).^47^ Estimated epithelial cell percentage per sample was extracted to be included as a covariate in our analysis.

### Statistical analysis

Three epigenome-wide analyses (EWA) were performed in this study: one with trimester 1’s PM_2.5_ average, one with trimester 2’s PM_2.5_ average, and one with the average PM_2.5_ over both trimesters. Each EWA was run using the *CpGassoc* package in R (R Core Team; Vienna, Austria). Linear models, adjusted for maternal age, maternal race, child sex, cell type composition, sample cohort, and row of sample of methylation array, were fit for association between a PM_2.5_ average and each of the CpG sites that passed quality control. We did not adjust for SES or related measures in this analysis, given the uniform low-income nature of our sample. Without meaningful variation in SES, there was no need for adjustment. Prior to the association, surrogate variable analysis (SVA) was performed to identify variation not attributed to previously identified biological and technical sources, and the surrogate variable was included in the model when observed *P* values otherwise greatly deviated from expected. After associations were run and *P* values generated, sites that passed the threshold for significance after adjustment via the false discovery rate (FDR) procedure were identified. Given that the relatively small sample size of our study could mask detection of statistically significant effects, we selected all CpG sites with *P* < 0.0001 for further study through pathway and network analysis.

Pathway and network analysis was performed using the Ingenuity Pathway Analysis (IPA) software (Qiagen; Venlo, Netherlands) that uses an expert level curated database of pathway interactions to generate putative pathways and networks. Input CpG sites were mapped to genes with both indirect and direct relationships to generate networks. The probability that the genes were included in the network not by chance was used to rank the networks by scores.^48^ If any networks overlapped, they were merged to create the largest possible network in order to maximize the number of physiological relationships that could be examined together. Canonical pathways associated with the genes regulated by input CpGs were identified with a ratio to examine pathway enrichment. They were also required to meet statistical significance through a *P* value calculated using a right-tailed Fisher exact test indicating the probability of the observed pathway association under a random model and adjusted for multiple testing. After the lists of sites with *P* < 0.0001 for each EWA were individually run through IPA pathway and network analysis, summaries were generated of major pathways and networks implicated by the genes in which those sites resided.

## Results

### Exclusions and sample characteristics

For this analysis, data were collected from 56 subjects in PRIDE and from 37 in ECSBio. Of these original 93 subjects, 91 were used in the final analysis. One subject was excluded due to an address that could not be geocoded precisely enough to assess air pollution. A second subject was excluded due to a mismatch between true sex and sex predicted from methylation patterns indicating unreliable methylation data. Table 1 details summary statistics for our sample of 91 mother-infant dyads. PM_2.5_ exposure, measured in μg/m^3^, averaged during trimester 1 had median 9.06, interquartile range (IQR) 8.47–10.36, and range 7.58–11.50. Exposure averaged during trimester 2 had median 8.15, IQR 7.89–8.75, and range 7.27–11.36, and exposure averaged over both trimesters 1 and 2 had median 8.71, IQR 8.45–9.30, and range 7.98–10.15.

**Table 1.** Study sample characteristics

Characteristics	Included subjects (n = 91)
Maternal factors	
Enrollment age (years); mean (SD)	22.6 (4.1)
Race, n (%)	
Black	50 (54.9)
White	33 (36.3)
Other	8 (8.8)
Child factors	
Sex; n (%)	
Female	42 (46.2)
Male	49 (53.8)
Gestational age at birth (weeks); mean (SD)	38.9 (1.6)

### Quality control

After probe-level and site-level quality control, 3,837 CpG sites were excluded for low bead counts, 221,486 were excluded with detection *P* > 0.01, and 55,081 were excluded due to locations on sex chromosomes, locations at or near SNP loci, or potential cross-hybridization. Following exclusions, a total of 585,604 CpG sites were available for epigenome-wide associations. The QQ plots from the EWA with trimester 1 (eFigure 1; http://links.lww.com/EE/A203), trimester 2 (see eFigure 3; http://links.lww.com/EE/A203), and the combined period (eFigure 5; http://links.lww.com/EE/A203) did not show large deviations between expected and observed p-values.

### Average prenatal PM_2.5_ exposure

**Figure 1.**
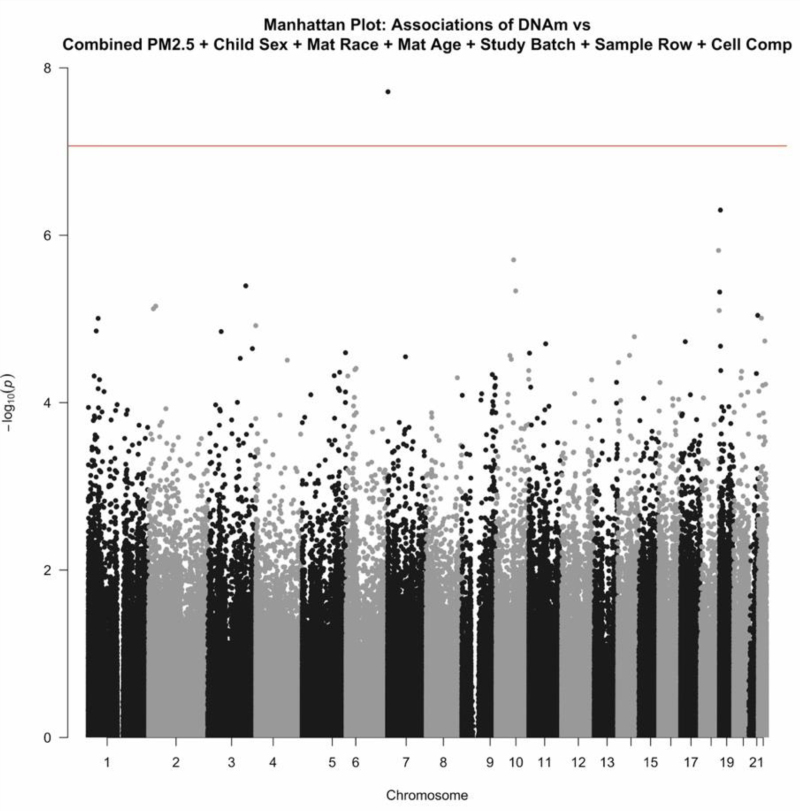
Manhattan plots for the association between DNA methylation at each studied CpG site and average PM_2.5_ over trimesters 1 and 2 of pregnancy adjusted for specified covariates. The vertical axis plots the negative log_10_ *P* value for each association. The Bonferroni cutoff for significance is denoted by the horizontal red line.

None of the CpG sites included in the analysis of PM_2.5_ averaged over trimester 1 were found to have DNAm significantly associated with average PM_2.5_ exposure in the first trimester after FDR correction (eFigure 2; http://links.lww.com/EE/A203). Similarly, DNAm was not associated with PM_2.5_ exposure averaged over trimester 2 (eFigure 4; http://links.lww.com/EE/A203). In the analysis of PM_2.5_ averaged over both trimester 1 and trimester 2, DNAm at one CpG site, cg18705808, was found to be significantly associated with PM_2.5_ exposures after FDR correction (Fig. 1). This site is found within the body of gene *TMEM184A*. The 10 sites with the lowest *P* values from each analysis are listed in Table 2.

**Table 2.** Ten CpG sites with the lowest *P* values from each EWA

Trimester 1						
CpG site	Chr	Gene	Location	M coefficient^c^	*P*	β coefficient (95% CI)^d^
cg14305641^a^	19	*MBD3*	3′ UTR	0.792	4.62 × 10^–07^	0.015 (0.008, 0.022)
cg16668903	5	*SNX24*	Body	0.714	6.83 × 10^–07^	0.013 (0.007, 0.018)
cg10940724	9	*DPP7*	TSS1500	1.615	1.82 × 10^–06^	0.142 (0.086, 0.198)
cg10419550	8	Intergenic	Intergenic	1.094	3.17 × 10^–06^	0.175 (0.109, 0.240)
cg13898459^a^	19	*PLIN4*	TSS1500	0.949	3.92 × 10^–06^	0.211 (0.134, 0.289)
cg16180217	3	*DCBLD2*	TSS200	0.598	4.56 × 10^–06^	0.132 (0.076, 0.188)
cg00672633	6	Intergenic	Intergenic	1.758	4.72 × 10^–06^	0.404 (0.241, 0.568)
cg25799969^a^	2	*PPP1CB*	5′ UTR; 1st Exon	1.758	6.38 × 10^–06^	0.167 (0.088, 0.246)
cg07309144	17	*NPEPPS*	Body	1.929	8.05 × 10^–06^	0.198 (0.111, 0.286)
cg02330710	1	*YTHDF2*	Body	1.414	8.59 × 10^–06^	0.343 (0.199, 0.486)
Trimester 2						
CpG site	Chr	Gene	Location	M coefficient^c^	*P*	β coefficient (95% CI)^d^
cg11845050	22	*PI4KAP2*	TSS200	0.766	1.30 × 10^–07^	0.033 (0.022, 0.044)
cg13804427	10	*PROSER2*	5′ UTR	0.931	6.73 × 10^–06^	0.056 (0.037, 0.075)
cg02397114	17	*TMEM102*	TSS200	0.673	7.72 × 10^–06^	0.023 (0.013, 0.032)
cg10180496	4	*UBE2D3*	TSS1500; 5′ UTR; TSS200; Body	1.450	8.83 × 10^–06^	0.101 (0.061, 0.142)
		*LOC105377348*	TSS1500			
cg10156714	20	*SLC2A10*	Body	0.635	8.99 × 10^–06^	0.024 (0.016, 0.032)
cg25967419	6	*KLC4*	TSS1500, TSS200	1.872	1.09 × 10^–05^	0.045 (0.029, 0.061)
		*MRPL2*	TSS200			
cg10149123	16	*C16orf91*	Body	–2.231	1.28 × 10^–05^	–0.277 (–0.377, –0.177)
cg16493531	6	*FLOT1*	TSS1500	–1.979	1.45 × 10^–05^	–0.424 (–0.580, –0.267)
		*IER3*	Body			
cg23528705	7	*UNCX*	Body	1.600	1.50 × 10^–05^	0.075 (0.059, 0.098)
cg02297831	4	*MED28*	TSS200	–1.948	1.55 × 10^–05^	–0.025 (–0.038, –0.013)
Trimesters 1 and 2 combined						
CpG site	Chr	Gene	Location	M coefficient^c^	*P*	β coefficient (95% CI)^d^
cg18705808^b^	7	*TMEM184A*	Body	1.375	1.93 × 10^–08^	0.025 (0.015, 0.036)
cg13898459^a^	19	*PLIN4*	TSS1500	11.017	5.00 × 10^–07^	1.328 (0.762, 1.893)
cg13852093	18	*MBP*	Body	16.052	1.52 × 10^–06^	2.189 (1.491, 2.887)
cg17706097	10	Intergenic	Intergenic	1.949	1.97 × 10^–06^	0.312 (0.182, 0.443)
cg11931463	3	Intergenic	Intergenic	1.981	4.02 × 10^–06^	0.411 (0.239, 0.584)
cg09308091	10	*ZMIZ1*	Body	1.572	4.62 × 10^–06^	0.301 (0.178, 0.424)
cg14305641^a^	19	*MBD3*	3′ UTR	3.704	4.77 × 10^–06^	0.192 (0.096, 0.288)
cg25799969^a^	2	*PPP1CB*	5′ UTR; 1st Exon	3.657	7.03 × 10^–06^	0.847 (0.540, 1.155)
cg27117509	2	*OSR1*	3′ UTR	3.479	7.55 × 10^–06^	0.181 (0.098, 0.265)
cg26020805	18	*CTDP1*	Body	2.950	7.94 × 10^–06^	0.249 (0.147, 0.351)

^a^Sites in both top 10 of trimester 1 and combined EWAs.

^b^Site found to be significant after FDR correction.

^c^Regression parameter from EWAs on DNAm m-value corresponding an increase in 10 μg/m^3^ of PM_2.5._

^d^Regression parameter from EWAs on DNAm β-value corresponding an increase in 10 μg/m^3^ of PM_2.5_ Pathway and Network Analysis.

CI indicates confidence interval.

After selecting only sites with unadjusted *P* < 0.0001, the trimester 1 EWA produced 62 sites, the trimester 2 EWA produced 80 sites, and the trimesters 1 and 2 EWA produced 82 sites. The following 10 genes were housing sites with *P* < 0.0001 in both the trimester 1 EWA and the trimesters 1 and 2 EWA: *SULT4A1*, *PPP1CB, TMEM184A, GEMIN5, MBD3, CTDP1, KCNQ4, PLIN4, MBP, and KCNQ1*. No genes from other EWAs overlapped with genes housing sites with *P* < 0.0001 in the trimester 2 EWA. Summaries of major pathways and networks identified by IPA pathway and network analysis are detailed in Table 3.

**Table 3.** Top pathways and networks enriched by genes regulated by top CpG sites using IPA

Trimester 1	
Top canonical pathways	–log_10_(*P*)
5-aminoimidazole ribonucleotide biosynthesis I	2.62
Purine nucleotides de novo biosynthesis II	2.06
DNA methylation and transcriptional repression signaling	1.56
Dermatan sulfate biosynthesis (late stages)	1.43
Chondroitin sulfate biosynthesis (late stages)	1.41
Top networks	Score
Cancer, cardiac necrosis/cell death, cell death and survival	23
Cancer, cellular compromise, organismal injury and abnormalities	23
Trimester 2	
Top canonical pathways	–log_10_(*P*)
Iron homeostasis signaling pathway	1.94
Human embryonic stem cell pluripotency	1.79
Circadian rhythm signaling	1.4
Complement system	1.37
G protein signaling mediated by tubby	1.3
Top networks	Score
Infectious diseases, cell-to-cell signaling and interaction, renal and urological system development and function	26
Cellular response to therapeutics, cell-to-cell signaling and interaction, nervous system development and function	20
Embryonic development, organismal development, gene expression	20
Trimesters 1 and 2	
Top canonical pathways	–log_10_(*P*)
Wnt/Ca+ pathway	2.63
Wound healing signaling pathway	2.59
Insulin secretion signaling pathway	2.5
Pulmonary fibrosis idiopathic signaling pathway	2.28
Neuropathic pain signaling in dorsal horn neurons	2.27
Top networks	Score
Lipid metabolism, small molecule biochemistry, cell cycle	27
Cell death and survival, connective tissue development and function, cellular movement	24
RNA damage and repair, protein synthesis, infectious diseases	4
Developmental disorder, hereditary disorder, ophthalmic disease	2

Scores for top networks represent a ranking of the networks least likely to be generated by chance with scores over 2 denoting a 99% likelihood of not being generated by chance.

## Discussion

In this study, DNAm at one CpG site in the body of the *TMEM184A* gene was significantly associated with PM_2.5_ averaged over the first two trimesters. No significant associations were observed between exposure averaged over trimesters 1 or 2 and DNAm. There was no overlap in the genes housing the 10 CpG sites with the lowest *P* values in trimester 1 and trimester 2. Additionally, IPA revealed very different pathways and networks implicated by the top CpG sites in each trimester.

The *TMEM184A* gene encodes for the transmembrane protein 184A (TMEM184A). This transmembrane protein is found in vascular smooth muscle cells and endothelial cells and is a key receptor for heparin in vascular cells.^49^ TMEM184A plays an essential role in heparin-induced anti-inflammatory activity in vascular endothelial cells.^50^ Although it is unclear whether this hypermethylation is mechanistically responsible for PM_2.5_-induced inflammation, the observed association between increased PM_2.5_ exposure and cg18705808 DNAm is consistent with previously established links between PM_2.5_ and inflammation.^2,51,52^

The observation that IPA produced different results when given top CpG sites from different periods could suggest that PM_2.5_ exposure during pregnancy has unique downstream physiological effects depending on the timing of exposure. A different set of pathways and networks were associated with trimester 1 PM_2.5_ exposure compared with trimester 2. This could suggest a timing-specific association between PM_2.5_ exposure during pregnancy and DNAm in offspring buccal epithelial cells. However, using IPA with CpG sites that do not pass the significance threshold is an imperfect investigation. There is a chance that the differences seen are simply a result of chance. An analysis of specific CpG sites of interest with better temporal resolution for PM_2.5_ exposure would help to elucidate this relationship.

In a similar analysis of 1,551 participants, Gruzieva et al^32^ identified 14 CpG sites associated with average prenatal PM_2.5_ exposure across pregnancy, a corollary to cumulative exposure similar to our two trimester average. The CpG sites found significant by their analysis did not overlap with any of the top sites from our study, in part due to their measurement of cord blood DNAm instead of buccal epithelial cell DNAm. Their notably larger sample size lends them greater power, explaining their ability to identify so many significant associations. However, our study also included trimester-specific windows of exposure while Gruzieva et al^32^ examined only a cumulative exposure model. Considering the association between prenatal PM_2.5_ and infant DNAm at specific CpG sites with exposure occurring during specific trimesters of pregnancy affords us a more detailed perspective on the effect of PM_2.5_ exposure on development. It is true that we did not find results in the individual trimester analyses, which passed the FDR threshold for significance, but a lack of significant findings does not strictly mean that there are no differences between the two analyses. The fact that the top sites are vastly different between the first and second trimester EWASs suggests to us that differences in the effect of PM_2.5_ exposure between trimesters may exist, and this possibility deserves further exploration.

Our study had some limitations. First, the small sample size limited our power and prevented more complex analyses. It also limits the external generalizability of our findings when considering populations with different PM_2.5_ concentrations and composition. Although ambient air pollution exposures used here do not represent personal exposures, it was the most appropriate measure that could be used to answer our research question without introducing confounding by personal activities, behaviors, and characteristics.^53^ Studying ambient versus personal air pollution concentrations will allow us to make inferences on potential primary prevention strategies and allowed us to study the impact of air pollution on the epigenome without conducting expensive and timely personal sampling. Lastly, averaging PM_2.5_ across a trimester may not capture fluctuations of exposure over time, which may have biologic relevance. It is possible that exposure to PM_2.5_ is only associated with DNAm changes during short windows of time or that exposure closer to birth during the third trimester has a greater effect on DNAm. Those effects may be missed when averaging the exposure over the trimester. Additionally, this method is not equipped to consider that PM_2.5_ concentrations during the selected window may be associated with concentrations outside the window and that the effect seen is truly caused by those concentrations that are not included in the average. Future analyses should use statistical methods that can utilize daily PM_2.5_ measurements to provide greater temporal resolution.

In summary, our EWA identified an association between PM_2.5_ exposure averaged over the first two trimesters of pregnancy with the hypermethylation of cg18705808, a CpG site located within the gene *TMEM184A*. The associated protein TMEM184A is a vital receptor in a heparin-induced anti-inflammatory pathway, a finding consistent with the links between PM_2.5_ and inflammation. Pathway and network analysis revealed largely different biological processes implicated by PM_2.5_ exposure in trimester 1 versus in trimester 2, which could suggest a timing-specific effect of prenatal PM_2.5_ exposure on infant DNAm. More research needs to be done with a larger sample size and utilizing an approach with greater temporal resolution to explore this proposed timing-specific effect in combination with established effects of long-term cumulative exposures.

## Conflicts of interest statement

The authors declare that they have no conflicts of interest with regard to the content of this report.

The results reported herein correspond to specific aims of grant R56MD013006 to investigator A.T.F. and K.B. from the National Institute of Minority Health and Health Disparities. T.B.M. was supported by the National Institutes of Health (NIH) R01 HL132344 and R01 HG011411 grants. This work was also supported by grants from the Cincinnati Children’s Hospital Medical Center including Academic Research Committee and Trustee awards to K.B. and A.T.F.

## Acknowledgments

We would like to thank the many families and facilitators involved in the PRIDE and ECS BioBank studies for their contributions to the data used in this study.

## Supplementary Material


