# Supplementary material for: Epigenome-wide association of neonatal methylation and trimester-specific prenatal PM2.5 exposure
Source: Environ Epidemiol. 2022 Oct 3;6(5):e227. doi: 10.1097/EE9.0000000000000227 (PMC9556110; doi:10.1097/EE9.0000000000000227)
Supplement: Supplementary file 1 [file ee9-6-e227-s001.docx]

Epigenome-Wide Association of Neonatal Methylation and Prenatal PM_2.5_ Exposure Suggests Timing-Specific Effects

Milan N. Parikh^1^, Cole Brokamp^1,2^, Erika Rasnick^1^, Lili Ding^1,2^, Tesfaye B. Mersha^2,3^, Katherine Bowers^1,2^, Alonzo T. Folger^1,2^

^1^ Division of Biostatistics and Epidemiology, Cincinnati Children's Hospital Medical Center, Cincinnati, OH, USA

^2^ Department of Pediatrics, University of Cincinnati College of Medicine, Cincinnati, OH, USA

^3^ Division of Asthma Research, Cincinnati Children’s Hospital Medical Center, Cincinnati, OH, USA

# Supplementary Materials

#### Average Prenatal PM_2.5_ Exposure

##### Trimester 1

Analyzing average PM_2.5_ concentrations over trimester 1 generated a genomic control factor of 0.941 and a QQ plot showing minimal deviation of the observed p-value distribution from the expected under the null hypothesis (eFigure 1). The SVA was not included in the model as it negatively impacted the QQ plot.


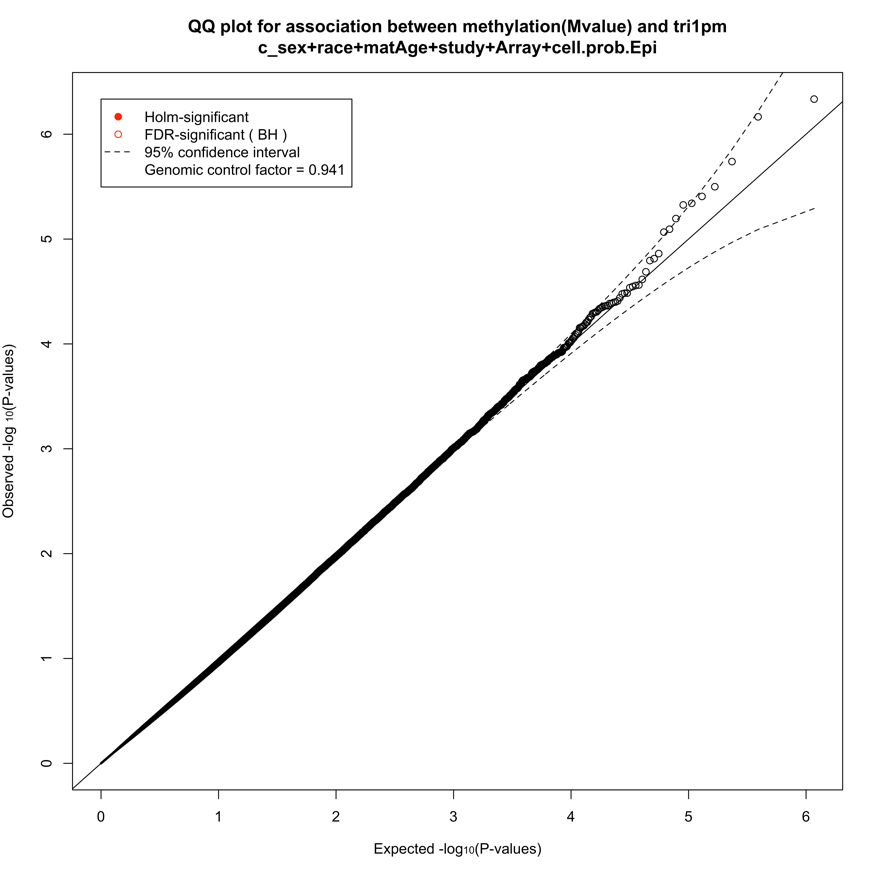


**eFigure 1.** QQ plot for the analysis between DNA methylation at each studied CpG site and PM_2.5_ averaged over trimester 1 of pregnancy with observed and expected p-values transformed on a negative log_10_ scale.


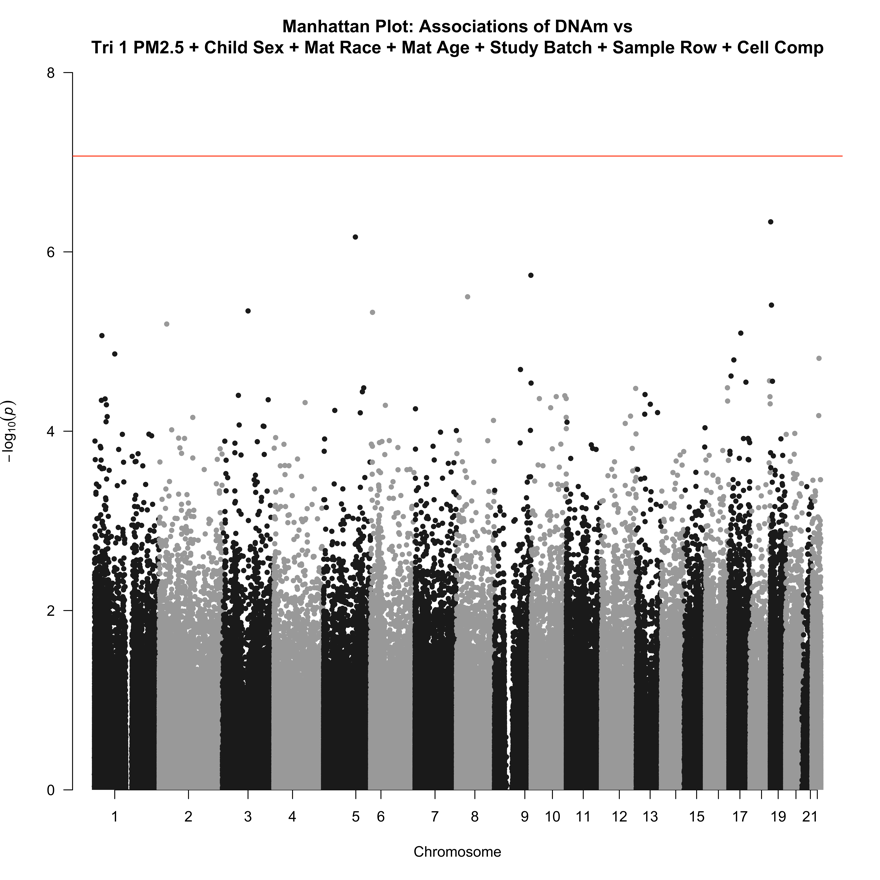


**eFigure 2.** Manhattan plots for the association between DNA methylation at each studied CpG site and PM_2.5_ averaged over trimester 1 of pregnancy adjusted for specified covariates. The vertical axis plots the negative log_10_ p-value for each association. The Bonferroni cutoff for significance is denoted by the horizontal red line.

##### Trimester 2

Analyzing average PM_2.5_ concentrations over trimester 2 generated a genomic control factor of 1.01 and a QQ plot with minimal deviations from expected (eFigure 3). This model was additionally adjusted for SVA as its inclusion greatly improved the QQ plot and the genomic control factor.


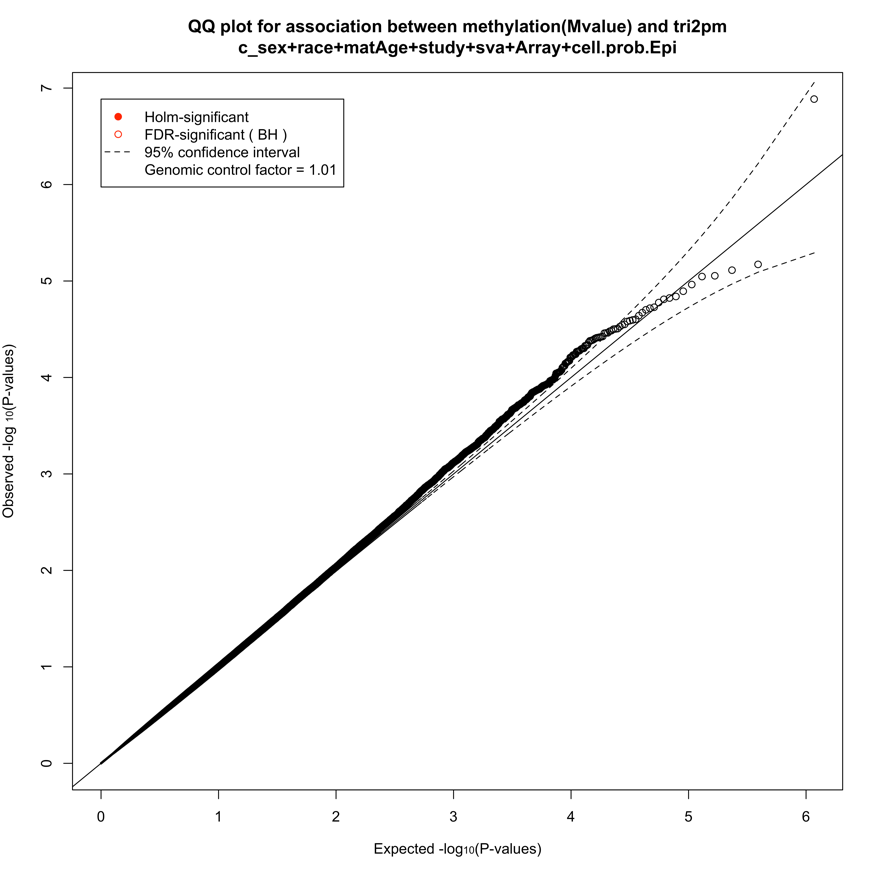


**eFigure 3.** QQ plot for the analysis between DNA methylation at each studied CpG site and PM_2.5_ averaged over trimester 2 of pregnancy with observed and expected p-values transformed on a negative log_10_ scale.


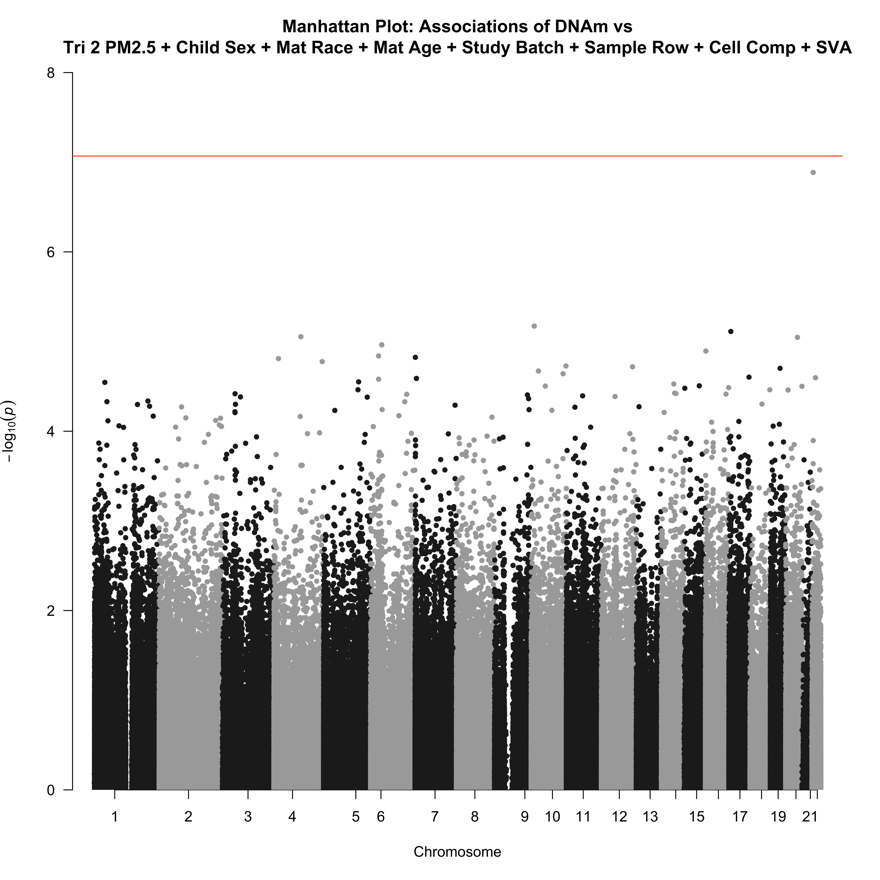


**eFigure 4.** Manhattan plots for the association between DNA methylation at each studied CpG site and PM_2.5_ averaged over trimester 2 of pregnancy adjusted for specified covariates. The vertical axis plots the negative log_10_ p-value for each association. The Bonferroni cutoff for significance is denoted by the horizontal red line.

##### Combined

Analyzing average PM_2.5_ concentrations across the first two trimesters generated a genomic control factor of 0.881 and a QQ plot with minimal deviations from expected (eFigure 5). The SVA was not included in the model as it negatively impacted the QQ plot.


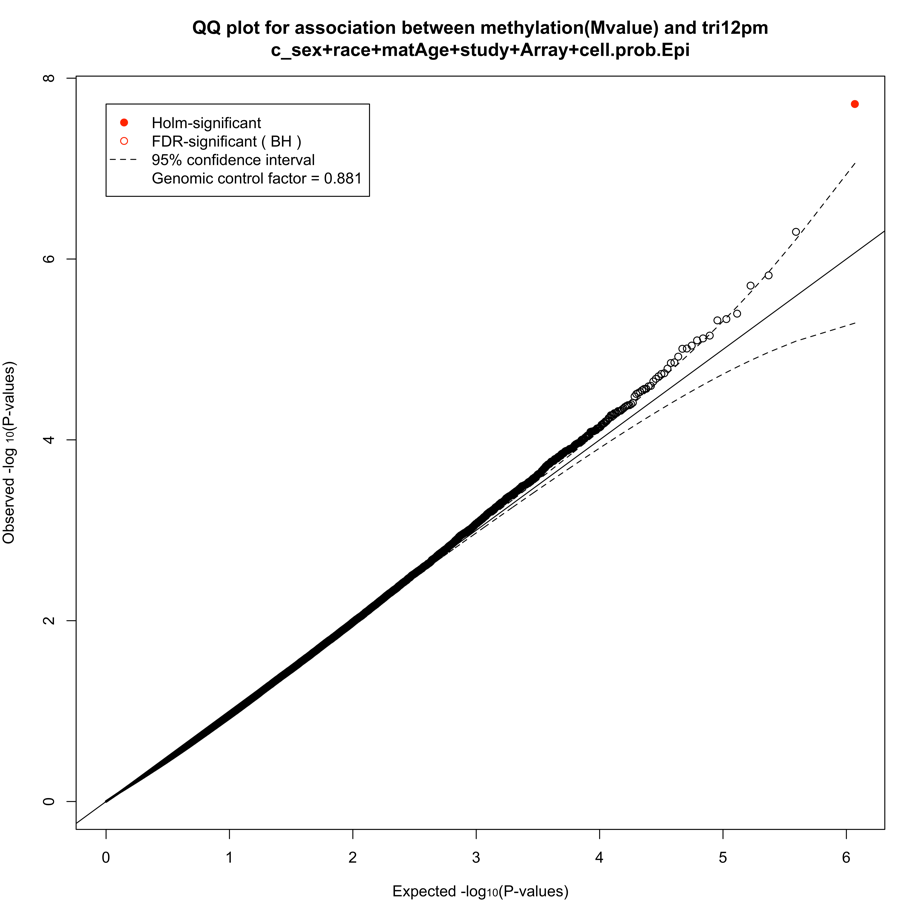


**eFigure 5.** QQ plot for the analysis between DNA methylation at each studied CpG site and PM_2.5_ averaged over trimesters 1 and 2 of pregnancy with observed and expected p-values transformed on a negative log_10_ scale.
